# Transcriptional Analysis Revealing the Improvement of ε-Poly-L-lysine Production from Intracellular ROS Elevation after *Botrytis cinerea* Induction

**DOI:** 10.3390/jof10050324

**Published:** 2024-04-29

**Authors:** Chen Zhang, Zhanyang Zhang, Ya Cheng, Ni Ni, Siyu Tong, Wangbao Da, Chunyan Liu, Qiran Diao, Ziyan Chen, Bingyue Xin, Huawei Zeng, Xin Zeng, Dayong Xu

**Affiliations:** 1 Anhui Province Key Laboratory of Pollutant Sensitive Materials and Environmental Remediation, Huaibei Normal University, Huaibei 235000, China; zc199910162023@163.com (C.Z.); zyzhang123123@163.com (Z.Z.); 18365587657@163.com (Y.C.); 19577398660@163.com (N.N.); t13637219368@163.com (S.T.); dawangbao2023@163.com (W.D.); 18133151637@163.com (C.L.); 18715262486@163.com (Q.D.); czy3024769527@163.com (Z.C.); xinbingyuex@163.com (B.X.); huaweizeng@163.com (H.Z.); 2 School of Life Sciences, Huaibei Normal University, Huaibei 235099, China

**Keywords:** biocontrol, *Botrytis cinerea*, ε-poly-L-lysine, reactive oxygen species, transcriptome analysis

## Abstract

Gray mold, caused by *Botrytis cinerea*, poses significant threats to various crops, while it can be remarkably inhibited by ε-poly-L-lysine (ε-PL). A previous study found that *B. cinerea* extracts could stimulate the ε-PL biosynthesis of *Streptomyces albulus*, while it is unclear whether the impact of the *B. cinerea* signal on ε-PL biosynthesis is direct or indirect. This study evaluated the role of elevated reactive oxygen species (ROS) in efficient ε-PL biosynthesis after *B. cinerea* induction, and its underlying mechanism was disclosed with a transcriptome analysis. The microbial call from *B. cinerea* could arouse ROS elevation in cells, which fall in a proper level that positively influenced the ε-PL biosynthesis. A systematic transcriptional analysis revealed that this proper dose of intracellular ROS could induce a global transcriptional promotion on key pathways in ε-PL biosynthesis, including the embden-meyerhof-parnas pathway, the pentose phosphate pathway, the tricarboxylic acid cycle, the diaminopimelic acid pathway, ε-PL accumulation, cell respiration, and energy synthesis, in which sigma factor HrdD and the transcriptional regulators of TcrA, TetR, FurA, and MerR might be involved. In addition, the intracellular ROS elevation also resulted in a global modification of secondary metabolite biosynthesis, highlighting the secondary signaling role of intracellular ROS in ε-PL production. This work disclosed the transcriptional mechanism of efficient ε-PL production that resulted from an intracellular ROS elevation after *B. cinerea* elicitors’ induction, which was of great significance in industrial ε-PL production as well as the biocontrol of gray mold disease.

## 1. Introduction

Gray mold, caused by *Botrytis cinerea*, is a plant disease that poses significant threats to various agricultural and horticultural crops [1,2,3,4]. After a period of winter dormancy, Botrytis cinerea is known to produce a substantial quantity of conidia during spring under increased humidity and temperature, which are dispersed through the air and droplets [3,4]. The infection of host tissues by airborne conidia involves sequential processes, including the secretion of extracellular enzymes (pectin methylesterases, polygalacturonases, laccases, and proteases), the degradation of cell walls, penetration by mycelia, colonization, and the eventual decay of tissues [5,6]. These extracellular enzymes exhibit differential expression patterns in response to different hosts and environmental factors, highlighting the broad range of hosts susceptible to *B. cinerea* infection [7,8,9]. If host plants become infected while in a latent state, favorable conditions will trigger the germination of B. cinerea conidia, leading to serious damage, particularly in stored and transported post-harvest fruits, such as widespread grape rot in the wine industry [10].

Despite the widespread use of chemical fungicides to address this issue, they have inherent drawbacks, such as the development of *B. cinerea* resistance, environmental pollution from chemicals, and potential health hazards [11]. In contrast to chemical fungicides, microbial antagonists offer better environmental and food safety profiles while effectively controlling plant pathogens; thus, they have been employed as biocontrol agents (BCAs) worldwide for managing plant diseases [12,13,14,15].

ε-Poly-L-lysine (ε-PL) is an antimicrobial agent produced by *Streptomyces* species through submerged fermentation. It is a natural cationic compound composed of 25 to 35 L-lysine residues that exhibits broad-spectrum antimicrobial activity against Gram-positive/negative bacteria, fungi, and even viruses [16]. With its high safety profile, good thermal stability, and water solubility characteristics, ε-PL has been extensively used as an efficient bio-preservative in Japan, Korea, EU countries, and the United States for several decades [17,18,19]. Generally speaking, ε-PL is believed to induce electrostatic interactions that disrupt cell membranes, leading to the disintegration of microbial cells [20,21,22]. Furthermore, ε-PL demonstrates excellent antimicrobial activity in the post-harvest preservation of fruits and vegetables, along with inhibitory effects on germ tube elongation and spore germination in *Alternaria alternata* [23] as well as gray mold rot caused by *Botrytis cinerea* [24,25,26]; hence, it represents a promising candidate for controlling plant microbial diseases.

In nature, the ε-PL-producing strain *Streptomyces albulus* IFO14147 (*S. albulus* IFO14147) exhibits slower growth rates compared to bacteria while displaying weaker abilities in terms of mycelial elongation and spore diffusion than molds. The complex natural environment compels *S. albulus* IFO14147 to confront other microorganisms with ε-PL, a chemical possessing broad-spectrum antimicrobial properties. Drawing inspiration from microbial interactions and competition, we hypothesized that ε-PL production would not be upregulated until an aggression signal is received from other microorganisms, suggesting that microbial signaling may play a crucial role in the biosynthesis of ε-PL. Our previous study demonstrated the positive impact of biomass extracts from *B. cinerea* on ε-PL biosynthesis [27]. However, it remains unclear whether the influence of *B. cinerea* signaling on ε-PL biosynthesis is direct or indirect.

In this study, we evaluated the microbial interaction between *B. cinerea* and *S. albulus* IFO14147 and observed an increase in intracellular ROS levels following induction by *B. cinerea* signaling during ε-PL production. Furthermore, we found that the induced ROS levels within an optimal range positively influenced ε-PL production, indicating that intracellular ROS serves as a mediating signal between *B.cinerea* signaling and enhanced ε-PL biosynthesis efficiency. Finally, a transcriptome analysis revealed insights into the underlying mechanism at the transcriptional level of genes involved in this process. This study provided evidence that elevated intracellular ROS induced by *B. cinerea* extracts acted as a mediating signal positively influencing ε-PL biosynthesis.

## 2. Materials and Methods

### 2.1. Strains and Culture Conditions

The ε-PL-producing strain, *S. albulus* IFO14147 (*S. albulus* CICC 11022), was purchased from the China Center of Industrial Culture Collection (CICC, Beijing, China), which was used throughout this study. The *B. cinerea* CGMCC 3.3790 was purchased from China General Microbiological Culture Collection Center (CGMCC, Beijing, China).

In this study, an agar slant medium was prepared for the cultivation of *S. albulus* IFO14147, *B. cinerea*, their interaction on solid medium, and the inhibition effect of ε-PL on mycelial growth of *B. cinerea*. This solid medium contained 10 g/L glucose, 5 g/L yeast extract, 5 g/L peptone, and 20 g/L agar with initial pH of 7.5 by adding 2 M NaOH. The spores of *S. albulus* IFO 14147 and *B. cinerea* were obtained after 7 days of cultivation on agar slant medium at 30 °C and 22 °C, respectively. For the interaction study between *S. albulus* IFO 14147 and *B. cinerea*, the spores of *S. albulus* IFO 14147 were inoculated at the central position of the agar slant medium and cultivated at 30 °C for 1 day, and then, the spores of *B. cinerea* were inoculated around the *S. albulus* colony and cultivated at 22 °C for 5 days. For the evaluation of ε-PL inhibition effects on *B. cinerea* mycelial growth, the *B. cinerea* spores were inoculated on the agar slant medium with ε-PL addition of 0, 200, 400, 600, 800, and 1000 μg/mL and cultivated at 22 °C for 5 days, after which the mycelial diameters of *B. cinerea* in different ε-PL concentrations were measured.

Medium 3G (M3G) was used for seed preculture and ε-PL production, and it was composed of 60 g/L glucose, 5 g/L yeast extract, 10 g/L (NH_4_)_2_SO_4_, 1.36 g/L KH_2_PO_4_, 0.8 g/L K_2_HPO_4_, 0.5 g/L MgSO_4_·7H_2_O, 0.04 g/L, ZnSO_4_·7H_2_O, and 0.03 g/L FeSO_4_·7H_2_O with an initial pH of 6.8. A modified YPD medium was used for submerged cultivation of *B. cinerea*, and it contained 40 g/L glucose, 10 g/L yeast extract, and 20 g/L peptone with an initial pH of 6.8. All these media were sterilized at 121 °C for 20 min, and the glucose was separately sterilized to prevent Maillard reaction. Seed preculture for ε-PL production was performed in 500 mL flasks with 60 mL presterilized M3G at 30 °C and 200 rpm for 1 day, which was initiated by 2 loops of spores (about 1 × 10^7^) for inoculation. ε-PL production was performed in a 250 mL flask with 30 mL working volume at 30 °C and 200 rpm for 2 days, which was initiated by adding 8% precultured seeds broth.

*B. cinerea* cultivation was carried out in a 500 mL flask with 80 mL working volume at 22 °C and 180 rpm for 4 days. The biomass was sequentially treated by centrifugation (5000× *g*), twice with water-rinsing, suspension in 70% ethanol, grinding in mortar with quartz sand, and centrifugation (5000× *g*). The supernatant was sterilized at 115 °C for 15 min and added in the ε-PL production as a mixture of *B. cinerea* elicitors at 24 h, after which the intracellular ROS dose was measured. The culture groups were set as CK (control check, 0 g wet cell/liter addition), LE (low dose of elicitors, 36 g wet cell/liter addition), and HE high dose of elicitors (72 g wet cell/liter addition).

To understand the effects of ROS on ε-PL production, H_2_O_2_ in different concentrations was added into the flasks at 24 h, and the intracellular ROS dose was measured at 26 h. The culture groups were set as CK (control check, 0 mM H_2_O_2_ addition), LOS (low oxidative stress, 30 mM H_2_O_2_ addition), and HOS (high oxidative stress, 70 mM H_2_O_2_ addition). In above ε-PL production, presterilized citric acid sodium buffer (pH 4.0) was added into the flasks at 24 h to maintain the constant pH of 3.8–4.3 with a final concentration of 10 g/L, and the ε-PL titer and dried cell weight (DCW) were measured.

### 2.2. Preparation of Sample for Transcriptome Analysis

The samples were taken from the flasks at 30 h in the CK, LOS, and HOS. Total RNA was prepared by centrifugation at 7000× *g* for 1 min, and precipitation was washed once, rapidly frozen in liquid nitrogen, and stored at −80 °C. The RNA isolation and RNA sequencing processes were carried out as described previously [9]. The related gene was mapped to the genome of *S. albulus* IFO14147 (CP104098.1). Only more than 2-fold differential transcripts [Log_2_^(EG/CK)^ > 1; *p*-value < 0.05] were considered significant, and these genes were selected. The transcription levels of genes in terms of cell reproduction, lipid catabolism, carbon source utilization, and secondary metabolite biosynthesis were investigated to understand the transcriptional influences of ROS on ε-PL production.

### 2.3. Transcriptional Analysis of Important Genes Related to ε-PL Biosynthesis

The key genes related to ε-PL biosynthesis were identified with quantitative real-time PCR (qRT-PCR). The transcriptional levels of genes (*zwf*, *N1H47_01765*, *N1H47_21215*, *N1H47_14640*, *N1H47_16885*, *N1H47_34205*, *N1H47_11760*, *N1H47_11815*, *N1H47_25630*, *N1H47_35560*) were measured with real-time fluorescent quantitative PCR (ABI Stepone plus, Applied Biosystems, Waltham, MA, USA) with SG Fast qPCR Master Mix (High Rox) (Bio Basic Inc., Toronto, ON, Canada). The sequence of primer pairs is given in Table S1.

### 2.4. Analytical Methods

The broth samples were centrifuged at 5000× *g* for 10 min, and the ε-PL titer in the supernatant and DCW in the sediment were determined as described in our previous study [28].

### 2.5. Statistical Analysis

For the effect evaluation of *B. cinerea* elicitors on intracellular ROS elevation and ε-PL production, the statistical analyses were performed using one-way analysis of variance (ANOVA) followed by an LSD (least significant difference) test with SPSS version 23.0 (SPSS, Inc., Chicago, IL, USA). All raw data were analyzed through 3 repeated tests, and the results were presented as means ± SD, in which significant differences (*p* < 0.05) and extremely significant differences (*p* < 0.001) were analyzed.

For the analysis of differential gene expression, the read counts from each sequenced library were adjusted using the edgeR program package through a scaling normalization factor. The differential expression analysis between two conditions was performed using the edgeR R package (version 3.24.3). The resulting *p*-values were adjusted using the Benjamini and Hochberg method. Significantly differentially expressed genes were defined as those with *padj* < 0.05 and |log_2_(foldchange)| > 1. The differential expression analysis of genes was performed using the clusterProfiler R package (version 3.8.1), which incorporated gene length bias correction. GO terms with a corrected *p* < 0.05 were considered significantly enriched by differentially expressed genes. ClusterProfiler R package was employed to test the statistical enrichment of differential expression genes in KEGG pathways. The figures were produced using GraphPad prism 9.0 (San Diego, CA, USA).

## 3. Results

### 3.1. Interaction between S. albulus IFO 14147 and B. cinerea

**Figure 1:**
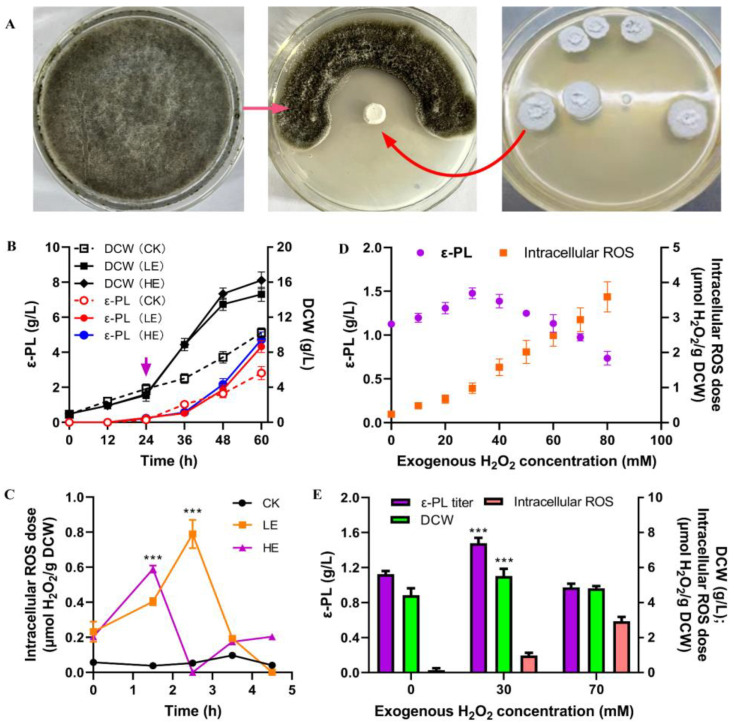
The mediate role of intracellular ROS in interactions between *S. albulus* and *B. cinerea*. (**A**) Interaction between *S. albulus* and *B. cinerea*. The arrow indicates the time of exogenous *B. cinerea* elicitors addition. (**B**) Improved ε-PL production after *B. cinerea* elicitors induction (CK, *B. cinerea* elicitors free; LE, 36 g wet cell/liter of elicitors addition; HE, 72 g wet cell/liter elicitors addition); (**C**) Intracellular ROS induction by *B. cinerea* elicitors; (**D**) Improved ε-PL production after exogenous H_2_O_2_ induction; (**E**) ε-PL production under different intracellular ROS doses. The results were presented as means ± SD, and the “***” indicates extremely significant differences (*p* < 0.001).

As depicted in Figure 1A, *S. albulus* IFO 14147 exhibited inhibitory effects on *B. cinerea* growth, primarily attributed to the antimicrobial agent ε-PL. Furthermore, media with elevated concentrations of ε-PL significantly reduced the mycelial diameter of *B. cinerea* (Table 1). Conversely, the supplementation of *B. cinerea* biomass extracts promoted the growth of *S. albulus* IFO 14147 and enhanced ε-PL production (Figure 1B). Therefore, ε-PL served as an effective biological weapon employed by *S. albulus* IFO 14147 against *B. cinerea* in its natural habitat; moreover, elicitors derived from this antagonistic biomass also induced improvements in cell growth and ε-PL biosynthesis rates. This intriguing phenomenon prompted us to elucidate its underlying mechanism. Figure 1C demonstrates a rapid increase in intracellular ROS levels, reaching a peak of approximately 0.6–0.8 μmol H_2_O_2_/g DCW after the addition of *B. cinerea* elicitors, both in the LE and HE conditions. This indicates that microbial elicitors can enhance transient intracellular ROS elevation, which may function as a secondary signal to regulate global transcriptional changes. To assess the mediating role of ROS between microbial call and ε-PL biosynthesis improvement, exogenous H_2_O_2_ was introduced during ε-PL production (Figure 1D,E). The addition of H_2_O_2_ within a range dose of 0–30 mM resulted in an enhancement of ε-PL production; however, higher doses (above 40 mM) had negative effects on this process. Specifically, the appropriate dosage (the LOS group with 30 mM H_2_O_2_ addition) led to a significant improvement of 27.5% in ε-PL production and a corresponding increase in cell growth by 24.7%. Conversely, high doses of H_2_O_2_ addition (the HOS group with 70 mM H_2_O_2_ addition) did not show any remarkable differences in either ε-PL production or cell growth rates. Notably, the level of intracellular ROS induced by *B. cinerea* elicitors (0.6–0.8 μmol H_2_O_2_/gDCW) closely resembled that observed in the LOS group (0.93 μmol H_2_O_2_/gDCW), suggesting that an appropriate level of intracellular ROS positively influences ε-PL production.

### 3.2. Transcriptome Analysis between Groups of LOS vs. CK and HOS vs. CK

**Figure 2:**
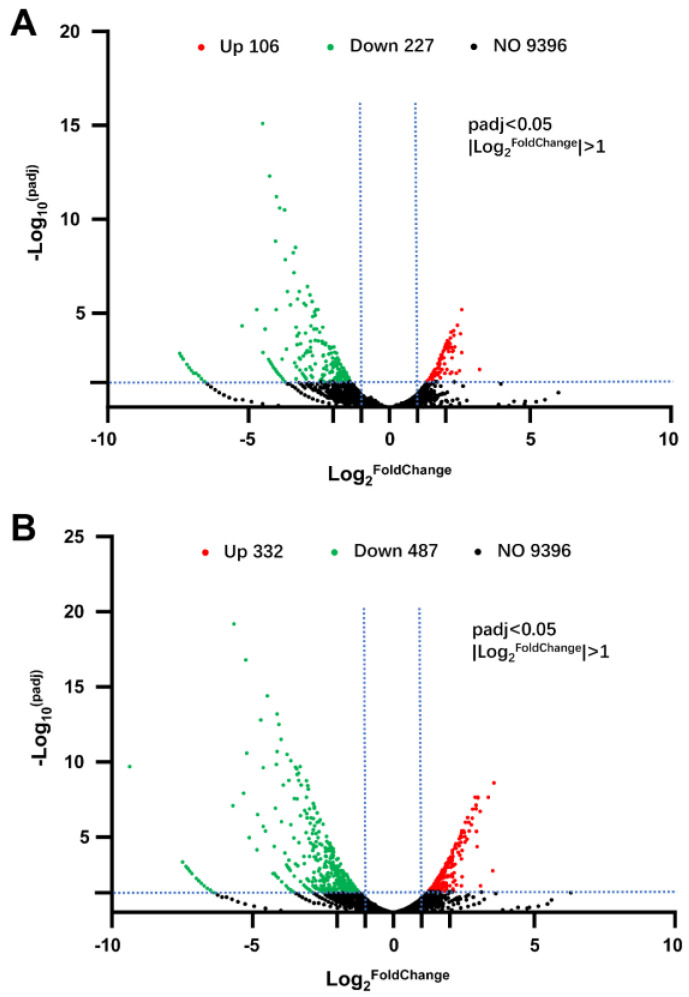
Interaction between Volcano diagrams showing the number of differentially expressed genes influenced by ROS induction. (**A**) LOS vs. CK; (**B**) HOS vs. CK. Up: upregulated DEGs, higher expression after intracellular ROS elevation; Down: downregulated DEGs, lower expression after intracellular ROS elevation; NO: no significant transcriptional difference after intracellular ROS elevation. The data are filtered under the standard of *padj* < 0.05 and |log_2_(foldchange)| > 1. The dashed line demarcates the genes exhibiting down-regulation, up-regulation, and no significant change in expression.

To elucidate the potential impact of elevated intracellular ROS on ε-PL production following *B. cinerea* elicitor induction, a transcriptome analysis and comparison were conducted among three groups: control (CK), low-dose oxidative stress (LOS), and high-dose oxidative stress (HOS). As depicted in Figure 2, LOS treatment resulted in the upregulation of 106 genes and downregulation of 227 genes, while HOS treatment led to the upregulation of 332 genes and downregulation of 487 genes. These findings indicate that an increase in intracellular ROS levels can significantly alter global gene transcription, with higher doses intensifying this effect.

**Figure 3:**
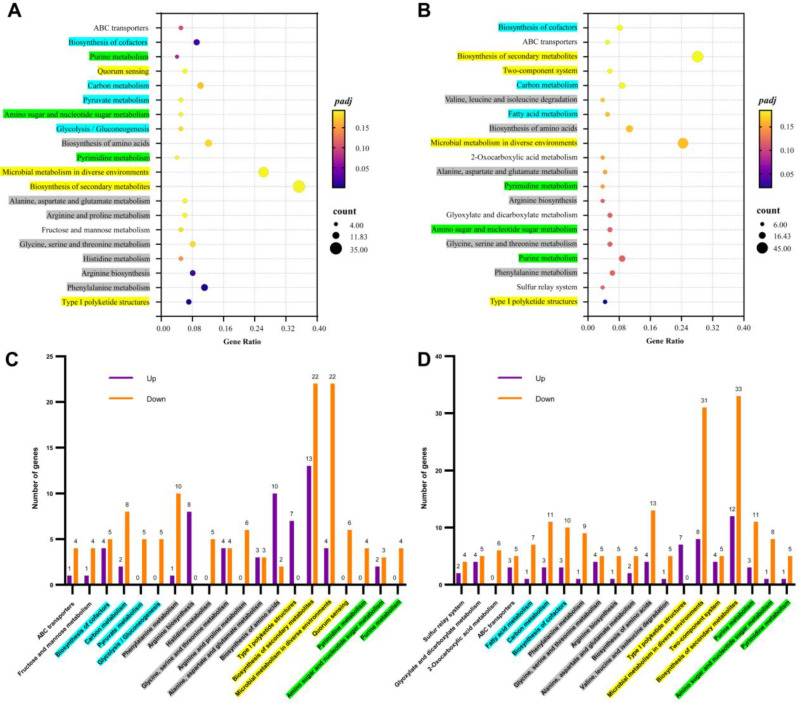
Influences of ROS on genes enriched in KEGG pathways (*padj* ≤ 0.05). Top 20 significantly enriched KEGG pathways in LOS vs. CK (**A**) or HOS vs. CK (**B**), in which the coordinate position and size of the dots (in **A**,**B**) indicate the ratio of DEGs and gene count involved in the KEGG term, and the color (in **A**,**B**) represents the *p*-value. Significantly enriched KEGG pathways in LOS vs. CK (**C**) and HOS vs. CK (**D**), in which the numbers of differentially expressed genes were summarized as Up (upregulated DEGs) and Down (upregulated DEGs) in different colors.

For the KEGG clustering analysis, the 20 most significant differentially expressed genes were involved in global carbon metabolism, amino acid metabolism, secondary metabolite biosynthesis, and nucleotide metabolism (Figure 3A,B). Compared with that in the CK, the LOS and HOS showed similar changes in gene transcription, while gene transcription was greatly changed in the HOS (excessive ROS) in terms of 2-oxocarboxylic acid metabolism, glyoxylate metabolism, dicarboxylate metabolism, and the sulfur relay system.

For the KEGG clustering analysis, the top 20 differentially expressed genes were primarily involved in global carbon metabolism, amino acid metabolism, secondary metabolite biosynthesis, and nucleotide metabolism (Figure 3A,B). Compared to the CK group, both LOS and HOS treatments exhibited similar changes in gene transcription; however, HOS treatment induced more pronounced alterations specifically related to 2-oxocarboxylic acid metabolism, glyoxylate metabolism, dicarboxylate metabolism, and sulfur relay system pathways. While most gene transcripts decreased due to elevated intracellular ROS levels (Figure 3C,D), a subset showed increased expression.

The elevation of intracellular ROS resulted in a decrease in the transcription of most genes, while some genes showed an increase in transcription (Figure 3C,D). In the LOS, upregulated genes were observed in arginine biosynthesis, amino acid biosynthesis, and type I polyketide structures. However, only genes related to type I polyketide structures were upregulated in the HOS (Figure 3C,D). Microbial metabolism genes in diverse environments and secondary metabolite biosynthesis exhibited significant changes in gene transcription, with ratios of 12/33 (up/down) and 13/22 (up/down) for LOS vs. CK as well as 8/31 and 4/22 for HOS vs. CK, respectively. These findings demonstrated that elevated ROS could induce overall transcriptional modifications on environmental adaptation and secondary metabolite biosynthesis. Notably, even though elevated intracellular ROS downregulated most genes involved in amino acid metabolism, those related to arginine biosynthesis were still upregulated under LOS conditions. This suggested that proper doses of ROS could improve arginine biosynthesis, which was beneficial for maintaining intracellular pH levels and cell survival under acidic culture conditions (pH 4.0) (Figure 1E).

**Figure 4:**
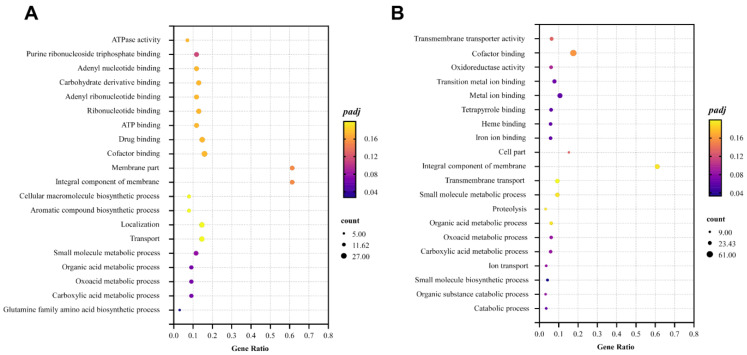
Top 20 significantly enriched Gene Ontology terms (*padj* ≤ 0.05) in LOS vs. CK (**A**) and HOS vs. CK (**B**), in which the coordinate position and size of the dots indicate the ratio of DEGs and gene count involved in the KEGG term, respectively, and the color represents the *padj*-value.

For the GO enrichment analysis, the top 5 significantly enriched GO terms in the LOS were ATPase activity, membrane components, integral components of the membrane, the cellular macromolecule biosynthetic process, and the aromatic compound biosynthetic process. In contrast, the HOS exhibited transmembrane transporter activity, cofactor binding, integral components of the membrane, transmembrane transport, and the small molecule metabolic process as its top 5 enriched GO terms (Figure 4). These findings suggest that elevated intracellular ROS can impact membrane structure and transport processes.

### 3.3. Transcriptional Influence of Elevated Intracellular ROS on Key Genes in ε-PL Synthesis Metabolism

**Figure 5:**
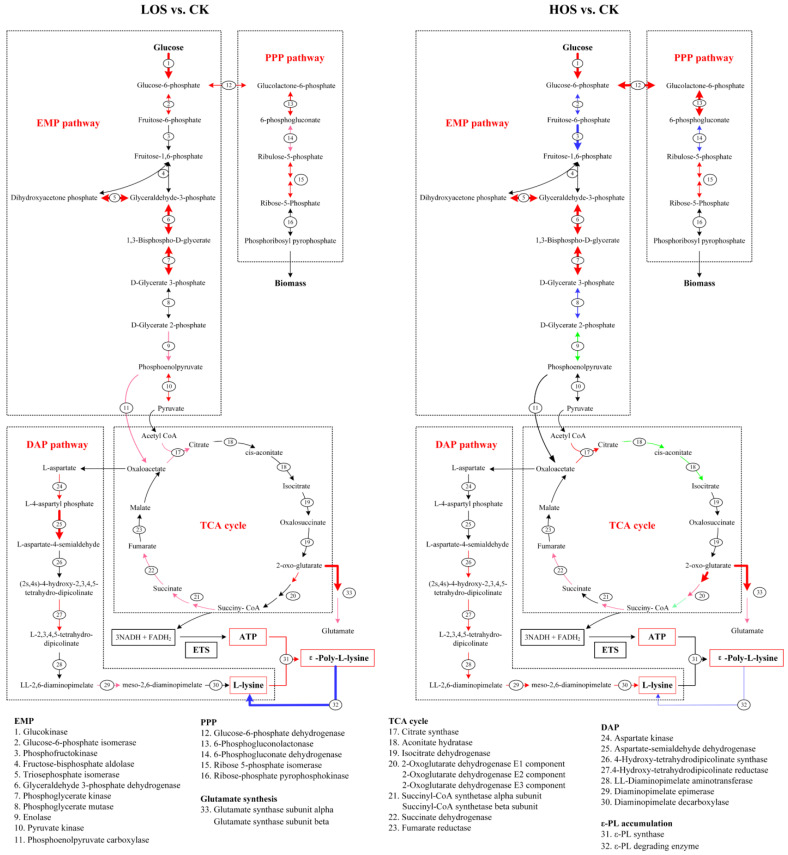
Transcription map analysis of genes related to ε-PL synthesis pathway (red thick arrow indicates gene upregulation by more than 2 times; red thin arrow indicates gene upregulation by 1.5 to 2 times; pink thin arrow indicates gene upregulation by 1.2 to 1.5 times; blue thick arrow indicates gene downregulation by less than 2 times; blue thin arrow indicates gene downregulation by 1.5 to 2 times; green thin arrow indicates gene downregulation by 1.2 to 1.5 times, and black arrow indicates gene difference is not significant).

The synthesis of ε-PL is highly reliant on the metabolic activities of the embden meyerhof pathway (EMP), pentose phosphate pathway (PPP), TCA cycle, and diaminopimelic acid pathway (DAP) [29]. To gain a comprehensive understanding of this phenomenon’s effects on key gene transcription within these pathways, the effects were summarized and analyzed (Table S2 and Figure 5).

In the EMP, an appropriate intracellular ROS dose in the LOS resulted in a more than 2-fold increase in transcriptional activity of genes encoding glucokinase (*N1H47_30055*), triosephosphate isomerase (*tpiA*), glyceraldehyde 3-phosphate dehydrogenase (*gap*), and phosphoglycerate kinase (*N1H47_10960*). Additionally, there was a 1.2–1.5-fold increase in gene encoding enolase (*eno*) and a 1.5–2.0-fold increase in gene encoding pyruvate kinase (*pyk*). These transcriptional enhancements ensured the availability of carbon skeletons for subsequent TCA cycle and DAP processes. However, excessive ROS negatively influenced gene transcription within the EMP. Notably, the gene encoding phosphofructokinase (*N1H47_11860*), which acts as a rate-limiting enzyme in the EMP, was downregulated in the HOS condition despite observed enhancements in genes encoding glucokinase (*N1H47_30055*), triosephosphate isomerase (*tpiA*), glyceraldehyde 3-phosphate dehydrogenase (*gap*), and phosphoglycerate kinase (*N1H47_10960*). This transcriptional inhibition of the rate-limiting enzyme gene would restrict metabolic flux through the EMP.

In the PPP, an appropriate intracellular ROS dose within the LOS led to higher levels of transcription for genes encoding glucose-6-phosphate dehydrogenase (*zwf*), 6-phosphogluconolactonase (*pgl*), and ribose 5-phosphate isomerase (*NIH47_14255*; increased by approximately 1.5–2.0-fold) as well as for the gene coding for 6-phosphogluconate dehydrogenase (gndA; increased by approximately 1.2–1.5-fold). However, excessive ROS dosage resulted in the downregulation of gndA gene expression by approximately 1.5–2.0-fold, while it still led to the upregulation of *zwf*, *pgl*, and NIH47_14255.

In the TCA cycle, an appropriate dosage of ROS in the LOS resulted in the increased transcription of genes encoding citrate synthase (*N1H47_14640*), 2-oxoglutarate dehydrogenase E1 component (*N1H47_26000*), succinyl-CoA synthase (*sucC*, *sucD*), and succinate dehydrogenase (*N1H47_11475*). The gene encoding phosphoenolpyruvate carboxylase (*N1H47_01765*) was up-regulated by 1.2- to 1.5-fold in the LOS, contributing to carbon skeleton supplementation for the TCA cycle. Although excessive intracellular ROS levels in the HOS led to a higher transcription of genes coding for citrate synthase (*N1H47_14640*), the 2-oxoglutarate dehydrogenase E1/E2 component (*N1H47_26000*, *sucB*), the succinyl-CoA synthetase alpha subunit (*sucD*), and succinate dehydrogenase (*N1H47_11475*), decreased transcription was observed in genes encoding aconitate hydratase (*acnA*) and the 2-oxoglutarate dehydrogenase E3 component (*lpdA*) at a fold change of approximately 1.2- to 1.5-fold. Interestingly, both LOS and HOS exhibited increased transcription levels of glutamate synthase genes (*gltB* and *N1H47_11385*), indicating that elevated intracellular ROS could promote glutamate synthesis as a key donor of NH_2_-groups for precursor L-lysine biosynthesis.

In DAP metabolism, an appropriate dosage of ROS in the LOS promoted the transcriptional activity of genes encoding aspartate kinase (*N1H47_21215*), 4-hydroxy-tetrahydrodipicolinate reductase (*dapB*), aspartate-semialdehyde dehydrogenase (*dapF*), and diaminopimelic acid epimerase (*dapF*). Similar improvements with more than a 150% increase were also observed in the HOS regarding gene expression related to 4-hydroxy-tetrahydrodipicolinate syntheses (*dapA*), 4-hydroxy-tetrahydropyridine reductase (*dapB*), diaminopimelate aminotransferase (*dapC*), diaminopimelate epimerase (*dapF*), and diaminopimelate decarboxylase (*lysA*). These findings indicated that metabolic activity within DAP can be positively influenced by elevated intracellular ROS.

Moreover, the appropriate dosage of ROS within the LOS significantly upregulated the transcription of the ε-PL synthetase gene (*pls*) by more than 2-fold and downregulated the transcription of the ε-PL-degrading enzyme gene (*pld*) by more than 2-fold, thereby promoting ε-PL accumulation. However, in the HOS, although *pld* transcription decreased, no significant improvement was observed in *pls* transcription. Consequently, efficient ε-PL accumulation occurred only with a proper intracellular dose of ROS.

In summary, the LOS (with an appropriate intracellular dose of ROS) exhibited consistent enhancement in gene transcription related to the EMP, the PPP, the TCA cycle, the DAP, and ε-PL accumulation, whereas excessive ROS within the HOS led to a metabolic disorder characterized by both the upregulation and downregulation of gene transcription involved in the EMP, PPP, and TCA cycle. This suggested that an optimal intracellular dose of ROS induced by *B. cinerea* elicitors could activate the ε-PL biosynthesis metabolism.

### 3.4. Transcriptional Influence of Elevated Intracellular ROS on Cell Respiration and Energy Production

As shown in Table S3, there were no significant differences observed between the LOS vs. CK and HOS vs. CK for complex I activity levels. However, compared to the CK group, the LOS showed higher transcript levels for genes associated with complex III (two out of two genes), complex IV (three out of three genes), and ATP synthase (seven out of eight genes), while less improvement was observed in transcript levels for these components within the HOS: complex III (one out of two genes), complex IV (two out three genes), and ATP synthase (three out eight genes). These findings suggested that an appropriate intracellular dose of ROS could enhance cellular respiration activity and energy production at the level of gene expression.

### 3.5. Transcriptional Influence of Elevated Intracellular ROS on Genes in Transcriptional Regulators and Secondary Metabolite Biosynthesis

Elevated intracellular ROS increased the transcription of genes coding for CynR (*cynR*) as well as transcriptional regulators in FurA (*N1H47_25630*) and LysR families (*N1H47_11470*, *N1H47_03465*, *N1H47_15470*, *N1H47_21305*), as shown in Table S4. Additionally, sigma factor HrdD, the positive transcriptional regulator of *pls*, exhibited increased transcription at an appropriate dose (in the LOS) and decreased transcription at excessive doses (in the HOS). Similar phenomena were observed in the transcriptional performance of genes coding for TetR (*N1H47_11760*) and MerR (*N1H47_11815*) family transcriptional regulators genes as well as TcrA (*N1H47_35560*), suggesting that these regulatory factors may participate in positively regulating elevated ROS in ε-PL production. Interestingly, intracellular ROS elevation not only modified gene expression related to ε-PL biosynthesis but also affected other secondary metabolite biosynthesis pathways involving at least 10 polyketide biosynthesis-related genes (Table S4). This indicated that ROS may be a crucial intracellular signal for the global regulation of secondary metabolite biosynthesis.

### 3.6. Transcriptional Verification of Key Genes in ε-PL Biosynthesis by qRT-PCR

**Figure 6:**
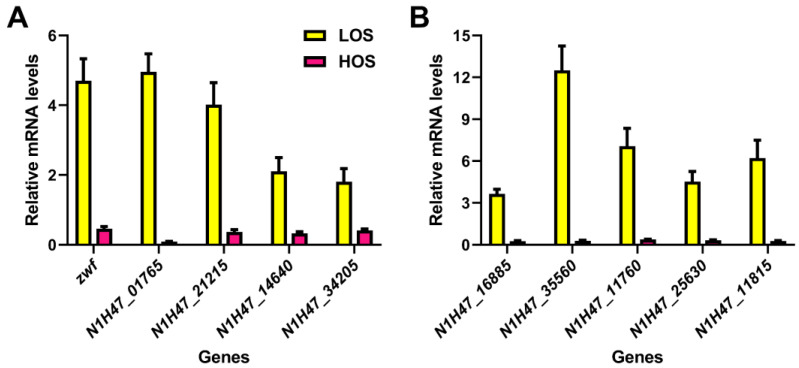
The effects of ROS induction on the transcription of key genes in ε-PL biosynthesis metabolism and relevant signal genes by qRT-PCR assays, where the gene transcription in the CK was set as 1, and those genes’ relative transcription in the LOS and HOS was calculated based on the levels of the CK. (**A**) Key genes in the ε-PL biosynthesis metabolism. (**B**) Important genes in relevant signal transduction.

The qRT-PCR assay was employed to validate the significant findings from the transcriptome analysis, including the transcription of key genes involved in ε-PL biosynthesis and transcriptional regulators. As depicted in Figure 6A, it was observed that LOS significantly upregulated the expression of genes encoding glucose-6-phosphate dehydrogenase (*zwf*), phosphoenolpyruvate carboxylase (*N1H47_01765*), aspartate kinase (*N1H47_21215*), citrate synthase (*N1H47_14640*), and Pls (*N1H47_34205*). This suggested that an appropriate level of ROS was beneficial for ε-PL biosynthesis. In Figure 6B, higher gene expression levels were detected for sigma factor HrdD (*N1H47_16885*) and transcriptional regulators TcrA (*N1H47_35560*), TetR (*N1H47_11760*), FurA (*N1H47_25630*), and MerR (*N1H47_11815*) in the LOS compared to the HOS. These results were consistent with the enhanced production of ε-PL and gene expression related to its biosynthesis under elevated intracellular ROS levels.

## 4. Discussion

The microbial interaction between *S. albulus* and *B. cinerea* established the basis for the biocontrol of plant gray mold. As a biocontrol agent secreted by *S. albulus*, ε-PL exhibited high inhibitory efficiency on the mycelial elongation of *B. cinerea* (Figure 1A and Table 1). Inspired by natural microbial interactions and competition, we proposed an innovative strategy to enhance ε-PL production by incorporating biomass extracts from *B. cinerea*, resulting in a 34% increase in ε-PL production [27]. However, it remained unclear whether the impact of microbial stress on ε-PL biosynthesis was direct or indirect.

**Figure 7:**
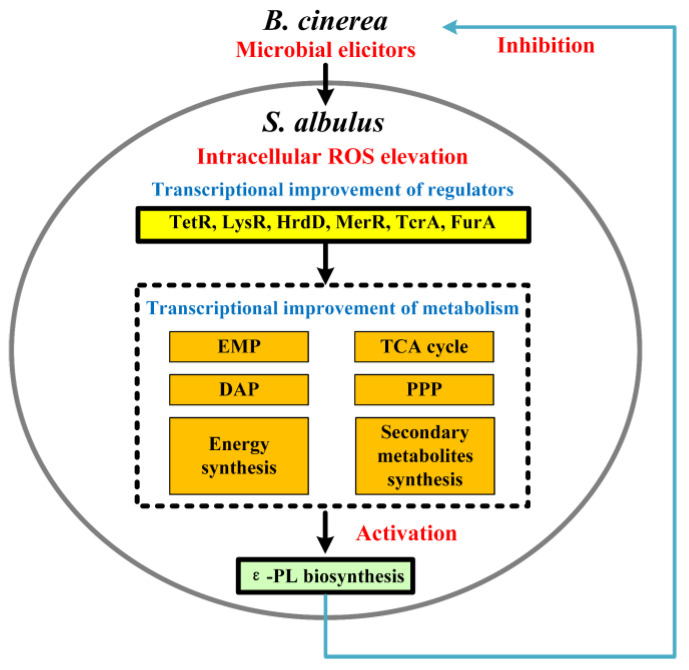
Putative mechanism of activated ε-PL production from elevated intracellular ROS after *B. cinerea* elicitors’ induction.

In this study, a remarkable elevation of intracellular ROS was observed after the *B. cinerea* elicitors’ induction (both in the LE and HE groups) (Figure 1C), which subsequently led to enhanced cell growth rate and ε-PL production (Figure 1B). As shown in Figure 7, a systematic transcriptional analysis revealed that the elevated ROS was in a proper dose that exactly induced a higher expression of several transcriptional regulators (HrdD and FurA-, LysR-, TetR-, MerR-family transcriptional regulators), which might be related to the global improvements of gene transcription in the EMP, the TCA cycle, the DAP, and glutamate synthase and *pls* genes, thereby enhancing carbon skeleton provision, energy production, precursor L-lysine synthesis, and ε-PL accumulation. Additionally, an optimal level of intracellular ROS-upregulated genes related to cell respiration and energy production, providing sufficient ATP for Pls-catalyzed L-lysine assembly. The global enhancement of ε-PL biosynthesis finally resulted in efficient ε-PL secretion, which could be used to reversely inhibit the elicitor host for better competition. Conversely, excessive ROS levels (in the HOS) disrupted gene transcription in these metabolic pathways. These conflicting changes in gene transcription hindered efficient ε-PL production by limiting carbon skeleton availability and energy supply. Interestingly, this increase in intracellular ROS not only enhanced ε-PL production but also altered gene transcription involved in the biosynthesis of other secondary metabolites, indicating that ROS served as a crucial intracellular signal for the global regulation of metabolism.

In fact, the induction of intracellular reactive oxygen species (ROS) has been reported to positively influence the production of secondary metabolites. For instance, in xanthan gum synthesis, elevated intracellular ROS induced by HClO can promote gene transcription and result in a 210% increase in xanthan gum yield and a 20% increase in viscosity [30]. Similarly, the transient elevation of ROS induced by H_2_O_2_ or heat-shock treatment can activate gene clusters involved in validamycin A synthesis, leading to an increased production of validamycin A [31,32]. Furthermore, natamycin biosynthesis can be enhanced by elevated ROS levels in a defective mutant lacking H_2_O_2_-detoxifying enzymes [33], which is attributed to metabolic promotion within the TCA cycle, respiratory chain, and branched-chain amino acid metabolism [34]. Additionally, appropriate levels of ROS have been found to promote carotene production and alter the composition of secondary metabolites [35]. Therefore, the modulation of the intracellular redox state plays a crucial role in secondary metabolite biosynthesis.

ROS exhibit high oxidative ability, enabling them to readily react with biomolecules within living cells. The prolonged elevation of ROS concentrations proves toxic to aerobic organisms; however, the transient induction of ROS at appropriate doses can serve as a signal to stimulate the biosynthesis of secondary metabolites in certain microorganisms, such as *Xanthomonas campestris* and *Streptomyces hygroscopicus* [30,31]. In this study, the elicitors from *B. cinerea* induced a temporary increase in intracellular ROS levels, which exerted global effects on metabolism and ε-PL production. Similar phenomena were observed during natamycin production by *S. natalensis* and rimocidin production by *S. rimosus* under fungal elicitor induction, resulting in elevated intracellular ROS levels and subsequent modifications in the transcriptional regulation of global genes, which are involved in branch-chained amino acid metabolism and specific secondary metabolite (natamycin and rimocidin) biosynthesis [36,37]. These findings imply that ROS acts as a secondary signal during microbial interactions, effectively activating *Streptomyces* species for enhanced antimicrobial agent production to facilitate a competitive advantage.

The present study demonstrated that intracellular ROS functioned as a secondary signaling molecule in the interaction between *B. cinerea* and *S. albulus* IFO14147, thereby activating the transcription of genes involved in ε-PL biosynthesis. This investigation enhanced our understanding of the mechanisms underlying enhanced ε-PL production following the “microbial call” by *B. cinerea*, thus providing a foundation for potential applications of ε-PL or live *S. albulus* in biocontrol strategies against gray mold disease.

## 5. Conclusions

The present study demonstrated that ROS functioned as an intracellular secondary signal mediating the induction of *B. cinerea* elicitors and efficient ε-PL production. The microbial call from *B. cinerea* triggered a controlled elevation of ROS levels in cells, which positively influenced the biosynthesis of ε-PL. Despite the downregulation of the majority of gene transcription by elevated ROS, an optimal dose of ROS induced global transcriptional promotion in key pathways involved in ε-PL biosynthesis, including the EMP, the PPP, the TCA cycle, the DAP, ε-PL accumulation, cell respiration, and energy synthesis. This global regulation involved Sigma factor HrdD and transcriptional regulators TcrA, TetR, FurA, and MerR. These findings provided valuable insights into the interaction mechanisms between molds and *Streptomyces* species, with significant implications for industrial ε-PL production and biocontrol strategies against gray mold disease.

## Figures and Tables

**Table 1:** Reduction of *B. cinerea* mycelial diameter under different ε-PL concentrations.

	ε-PL Concentration (μg/mL) ^a^					
	0	200	400	600	800	1000
Mycelial diameter (cm)	7.88 ± 0.72	6.45 ± 0.51	3.57 ± 0.33	1.89 ± 0.23	0.54 ± 0.08	0.17 ± 0.02

^a^ Data indicate the mycelial diameter of *Botrytis cinerea* cultivated for 5 days in petri plate with ε-PL addition of different concentrations.

## Data Availability

Data are contained within the article.

## Supplementary Materials

The following supporting information can be downloaded at https://www.mdpi.com/article/10.3390/jof10050324/s1, Table S1: Primer pairs sequences for quantitative real-time PCR (qRT-PCR) assay; Table S2: Transcriptome analysis of genes coding for enzymes in ε-PL biosynthesis pathways among cells with different intracellular ROS levels; Table S3: Influences of intracellular ROS levels on the genes’ transcription concerning energy production; Table S4: Influences of intracellular ROS levels on the genes’ transcription concerning transcriptional regulator and secondary metabolite biosynthesis.
