# Supplementary material for: Transcriptional Analysis Revealing the Improvement of ε-Poly-L-lysine Production from Intracellular ROS Elevation after Botrytis cinerea Induction
Source: J Fungi (Basel). 2024 Apr 29;10(5):324. doi: 10.3390/jof10050324 (PMC11122054; doi:10.3390/jof10050324)
Supplement: Supplementary file 1 [file jof-10-00324-s001.zip › jof-2949385-supplementary.pdf]

Transcriptional Analysis Revealing the Improvement of ε-Poly-L-lysine Production from Intracellular ROS Elevation after *Botrytis cinerea* Induction

Chen Zhang [1,2,†], Zhanyang Zhang [1,2,†], Ya Cheng [1,2], Ni Ni [1,2], Siyu Tong [1,2], Wangbao Da [1,2], Chunyan Liu [1,2], Qiran Diao [1,2], Ziyan Chen [1,2], Bingyue Xin [1,2], Huawei Zeng [1,2], Xin Zeng [1,2,*] and Dayong Xu [1,2,*]

1 Anhui Province Key Laboratory of Pollutant Sensitive Materials and Environmental Remediation, Huaibei Normal University, Huaibei 235000, China; zc199910162023@163.com (C.Z.); zyzhang123123@163.com (Z.Z.); 18365587657@163.com (Y.C.); 19577398660@163.com (N.N.); t13637219368@163.com (S.T.); dawangbao2023@163.com (W.D.); 18133151637@163.com (C.L.); 18715262486@163.com (Q.D.); czy3024769527@163.com (Z.C.); xinbingyuex@163.com (B.X.); huaweizeng@163.com (H.Z.)

2 School of Life Sciences, Huaibei Normal University, Huaibei 235099, China

* Correspondence: cengx@chnu.edu.cn (X.Z.); xudy@chnu.edu.cn (D.X.)

† These authors contributed equally to this work. .

Table S1 Primer pairs sequences for quantitative real-time PCR (qRT-PCR) assay.

Target gene	Primer name	Primer sequence (5′-3′)
16s rRNA	16s rRNA-forward	GCACAAGCAGCGGAGCAT
	16s rRNA -reverse	CCCAACATCTCACGACACGA
zwf	*zwf* -forward	CGTCCACGAGGTCTTCCC
	zwf -reverse	GGAGGAGGTGGTTCTGGATG
N1H47_01765	*N1H47_01765* -forward	CTAACTCAGCAGGCACTGTGTC
	N1H47_01765 -reverse	GACGTGGTAGGCGTTCTCC
N1H47_21215	*N1H47_21215* -forward	GTCCGCTCCTCCTTCTCG
	N1H47_21215 -reverse	CCGACGACCGTGACCTTC
N1H47_14640	*N1H47_14640* -forward	GCCTTCAAGCAGGACATCAC
	N1H47_14640 -reverse	GGTTGTGGCTGTCCTGGTAG
N1H47_16885	*N1H47_16885* -forward	GAGCTGTCCCAGACCATCG
	N1H47_16885 -reverse	GTTGGACCGGATGAAGACG
N1H47_34205	*N1H47_34205* -forward	CCCTGTGGTCGTCGTTCG
	N1H47_34205 -reverse	GAAGAGGTGGGTCTGCAGGA
N1H47_11760	*N1H47_11760* -forward	ACCGTGTACTACAACTTCGCG
	N1H47_11760 -reverse	ACGTACAGCTGGGTGAGGG
N1H47_11815	*N1H47_11815* -forward	AGGTGCTGCCCTGTCTGC
	N1H47_11815 -reverse	GTTCCGGGAGGCCATCAG
N1H47_25630	*N1H47_25630* -forward	CACTACACCGCCGACGAA
	N1H47_25630 -reverse	AGGGTGTTGTAGACCGTGGC
N1H47_35560	*N1H47_35560* -forward	CCTCATGGTGCTCGACGTC
	N1H47_35560 -reverse	CGGAAGGTAGTCGTCGGC

Table S2 Transcriptome analysis of genes coding for enzymes in ε-PL biosynthesis pathways among cells with different intracellular ROS levels.

Reaction	Gene ID	CK fpkm	LOS fpkm	HOS fpkm	Gene description
**EMP**					
1	*N1H47_30055*	35.68	76.51	86.51	Glucokinase
2	*pgi*	43.83	81.58	28.62	Glucose-6-phosphate isomerase
3	*N1H47_11860*	13.44	12.35	4.53	Phosphofructokinase
4	*fbaA*	32.76	30.24	31.22	Fructose-bisphosphate aldolase
5	*tpiA*	21.48	74.82	45.95	Triosephosphate isomerase
6	*gap*	38.84	122.32	83.42	Glyceraldehyde 3-phosphate dehydrogenase
7	*N1H47_10960*	39.26	112.01	90.60	Phosphoglycerate kinase
8	*N1H47_22570*	2287.06	2590.98	1256.65	Phosphoglycerate mutase
9	*eno*	51.85	71.29	36.87	Enolase
10	*pyk*	34.48	60.91	38.46	Pyruvate kinase
11	*N1H47_01765*	7.45	9.58	10.98	Phosphoenolpyruvate carboxylase
**PPP**					
12	*zwf*	123.19	232.08	295.68	Glucose-6-phosphate dehydrogenase
13	*pgl*	61.04	93.14	127.12	6-phosphogluconolactonase
14	*gndA*	676.42	838.07	400.21	Gluconate-6-phosphate dehydrogenase
15	*N1H47_14255*	745.10	1208.97	1255.08	Ribose 5-phosphate isomerase
16	*N1H47_16385*	38.30	34.64	37.65	Ribose-phosphate pyrophosphokinase
**TCA cycle**					
17	*N1H47_14640*	117.99	144.13	218.72	Citrate synthase
18	*acnA*	117.37	123.09	89.84	Aconitate hydratase
19	*N1H47_08375*	84.71	73.69	85.14	Isocitrate dehydrogenase

20	*N1H47_26000*	15.12	14.60	20.91	Oxoglutarate dehydrogenase E1 component
	sucB	29.74	29.21	24.00	Oxoglutarate dehydrogenase E2 component
	lpdA	148.34	194.07	127.39	Oxoglutarate dehydrogenase E3 component
21	*sucD*	261.14	320.37	320.12	Succinyl-CoA synthetase subunit alpha
	sucC	153.98	208.61	147.58	Succinyl-CoA synthetase subunit beta
22	*N1H47_11475*	68.92	100.37	87.38	Succinate dehydrogenase
23	*N1H47_24965*	36.56	35.02	37.94	Fumarate hydratase
**DAP**					
26	*N1H47_21215*	58.99	93.25	60.88	Aspartate kinase
27	*N1H47_21220*	58.11	125.03	69.30	Aspartate-semialdehyde dehydrogenase
28	*dapA*	18.51	18.10	10.48	4-Hydroxy-tetrahydrodipicolinate synthase
29	*dapB*	57.58	67.08	98.66	4-Hydroxy-tetrahydrodipicolinate reductase
30	*dapC*	31.54	54.96	58.14	Diaminopimelate aminotransferase
31	*dapF*	42.09	28.64	27.39	Diaminopimelate epimerase
32	*lysA*	49.84	58.61	77.04	Diaminopimelate decarboxylase
**ε-PL accumulation**					
33	*N1H47_34205*	23.66	41.01	22.83	ε-PL synthase
34	*N1H47_32795*	5.24	1.22	2.81	ε-PL degrading enzyme
**Glutamate synthesis**					
35	*gltB*	55.94	115.84	120.83	Glutamate synthase subunit alpha
	N1H47_11385	167.98	212.73	250.29	Glutamate synthase subunit beta

Table S3 Influences of intracellular ROS levels on the genes' transcription concerning energy production.

Gene ID	CK fpkm	LOS fpkm	HOS fpkm	Gene description
**Complex Ⅰ**				
N1H47_17850	55.30	39.53	3.62	NADH dehydrogenase subunit A
N1H47_17990	15.86	12.75	23.38	NADH dehydrogenase subunit B
N1H47_18410	2903.41	3248.27	4703.27	NADH dehydrogenase subunit C
N1H47_17980	9.94	7.97	10.00	NADH dehydrogenase subunit D
nuoE	22.86	19.61	26.97	NADH dehydrogenase subunit E
nuoF	85.38	79.65	163.66	NADH dehydrogenase subunit F
N1H47_17965	56.85	70.90	148.05	NADH dehydrogenase subunit G
nuoI	198.62	202.25	182.04	NADH dehydrogenase subunit I
N1H47_17950	114.09	132.22	159.97	NADH dehydrogenase subunit J
nuoK	496.33	373.54	565.08	NADH dehydrogenase subunit K
nuoL	98.61	81.28	122.15	NADH dehydrogenase subunit L
N1H47_17935	218.47	257.69	322.13	NADH dehydrogenase subunit M
nuoN	71.38	120.92	99.12	NADH dehydrogenase subunit N
N1H47_17835	140.44	172.43	139.43	NADH dehydrogenase subunit NuoH2
N1H47_17830	52.23	49.52	47.67	NADH dehydrogenase subunit NuoI2
**Complex Ⅲ**				
N1H47_11985	1405.11	2187.52	1333.84	Ubiquinol-cytochrome C reductase iron-sulfur 'Rieske' subunit
N1H47_11980	29.44	48.86	57.38	Menaquinol-cytochrome C reductase cytochrome b subunit
**Complex Ⅳ**				
ctaD	58.72	74.40	53.18	Cytochrome c oxidase subunit I
coxB	3.32	8.55	11.75	Cytochrome c oxidase subunit II
N1H47_11995	2047.29	3098.15	3875.01	Cytochrome c oxidase subunit III
**ATP synthase**				
atpA	359.88	443.50	329.43	ATP synthase subunit alpha
atpD	9.79	9.80	6.84	ATP synthase subunit beta
N1H47_26255	3.69	6.14	0.97	ATP synthase subunit gamma
N1H47_26245	219.96	251.91	294.54	ATP synthase subunit delta
N1H47_26265	2.58	4.34	1.18	ATP synthase subunit epsilon
atpB	7.30	22.69	13.38	ATP synthase subunit A
N1H47_26240	57.00	72.40	95.18	ATP synthase subunit B
atpE	21.53	66.92	24.76	ATP synthase subunit C

Table S4 Influences of intracellular ROS levels on the genes' transcription concerning transcriptional regulator and secondary metabolites biosynthesis.

**Gene ID**	**CK fpkm**	**LOS fpkm**	**HOS fpkm**	**Gene description**
**Transcriptional regulators**				
cynR	508.97	895.42	784.97	Transcriptional regulator CynR
N1H47_25630	299.81	868.58	706.28	FurA family transcriptional regulator
N1H47_11470	422.80	832.65	702.49	LysR family transcriptional regulator
N1H47_03465	364.00	548.44	1124.80	LysR family transcriptional regulator
N1H47_15470	257.71	315.49	707.20	LysR family transcriptional regulator
N1H47_21305	35.57	54.10	100.45	LysR family transcriptional regulator
N1H47_16885	6.34	8.73	3.45	Sigma factor HrdD
N1H47_11760	17.20	101.83	8.70	TetR family transcriptional regulator
N1H47_35560	31.40	110.69	17.18	Transcriptional regulatory protein TcrA
N1H47_11815	34.19	177.38	17.65	MerR family transcriptional regulator
**Biosynthesis of other secondary metabolites**				
N1H47_36795	104.25	181.19	184.46	Erythromycin esterase family protein
N1H47_05775	306.07	486.94	471.45	Type I polyketide synthase
N1H47_38515	204.50	374.19	489.09	Type I polyketide synthase
N1H47_05765	178.15	232.59	212.45	Type I polyketide synthase
N1H47_28655	96.21	193.67	170.80	Type III polyketide synthase
N1H47_05755	150.89	250.20	187.16	Type I polyketide synthase
N1H47_05780	68.48	132.78	143.85	Type I polyketide synthase
N1H47_38485	21.01	2.70	4.96	Type I polyketide synthase
N1H47_36780	23.33	10.01	5.50	Polyketide synthase
N1H47_01840	10.05	1.44	2.64	Acyl transferase in polyketide synthase
N1H47_31990	14.80	7.97	4.47	Beta-ketoacyl synthase
N1H47_00660	37.93	16.92	11.33	Type I polyketide synthase
